# Fast and Inexpensive Phenotyping and Genotyping Methods for Evaluation of Barley Mutant Population

**DOI:** 10.3390/plants9091153

**Published:** 2020-09-06

**Authors:** Yudai Kawamoto, Hirotaka Toda, Hiroshi Inoue, Kappei Kobayashi, Naoto Yamaoka, Takuya Araki, Takashi Yaeno

**Affiliations:** Department of Agriculture, Ehime University, Tarumi, Matsuyama, Ehime 790-8566, Japan; c610060y@mails.cc.ehime-u.ac.jp (Y.K.); f651010z@mails.cc.ehime-u.ac.jp (H.T.); c610024b@mails.cc.ehime-u.ac.jp (H.I.); kobayashi.kappei.mk@ehime-u.ac.jp (K.K.); yamaokan@agr.ehime-u.ac.jp (N.Y.); araki@agr.ehime-u.ac.jp (T.A.)

**Keywords:** barley, TILLING, waxy, CELI

## Abstract

To further develop barley breeding and genetics, more information on gene functions based on the analysis of the mutants of each gene is needed. However, the mutant resources are not as well developed as the model plants, such as Arabidopsis and rice. Although genome editing techniques have been able to generate mutants, it is not yet an effective method as it can only be used to transform a limited number of cultivars. Here, we developed a mutant population using ‘Mannenboshi’, which produces good quality grains with high yields but is susceptible to disease, to establish a Targeting Induced Local Lesions IN Genomes (TILLING) system that can isolate mutants in a high-throughput manner. To evaluate the availability of the prepared 8043 M_3_ lines, we investigated the frequency of mutant occurrence using a rapid, visually detectable waxy phenotype as an indicator. Four mutants were isolated and single nucleotide polymorphisms (SNPs) were identified in the *Waxy* gene as novel alleles. It was confirmed that the mutations could be easily detected using the mismatch endonuclease CELI, revealing that a sufficient number of mutants could be rapidly isolated from our TILLING population.

## 1. Introduction

Barley (*Hordeum vulgare* L.) is one of the most important cereal crops and is also essential as a model crop for staple cereals with complex genomic structures, such as polyploid durum and bread wheat. For the breeding of crops that can adapt to the rapidly changing environment in the future, high yield and quality are required, as well as resistance to diseases such as powdery mildew, Fusarium head blight (FHB), blast, and so on. Phenotypic and genomic information and gene expression profiles derived from numerous barley genotypes have been accumulated, but are yet insufficient to elucidate the functions of the genes themselves and the interactions between the associated genes. Indeed, the fact that only a limited number of cultivars, e.g., ‘Golden Promise’, can apply the transformation technique makes it difficult to analyze the correlation between phenotypes and genes specific to a particular cultivar [1]. For example, ‘Golden Promise’ carries the resistance gene *Mla8* against powdery mildew races that harbor the corresponding *AVRa8* gene, which causes cell death and makes it difficult to analyze other gene functions and molecular responses that are not directly related to *Mla8*. On the other hand, the dilemma is that cultivars that do not have the resistance gene cannot be transformed. Therefore, like model plants such as *Arabidopsis*, ideally, there should be a wide variety of genetic material of all cultivars for each purpose, and it is hoped that there will be a more readily available method of preparing the genetic resources of each cultivar in a shorter period of time and at a lower cost.

TILLING (Targeting Induced Local Lesions IN Genomes) is a high-throughput reverse genetic technique that allows for the isolation of a large number of allelic variants of candidate genes [2,3]. Originally, TILLING was developed as a tool for analyzing the gene function of model organisms, but now it has been applied to crop breeding. One of the features of TILLING is that it can save time, cost, and space by simultaneously screening many individuals for the gene of interest. It is less sensitive to genome size and polyploidy and thus can be applied to a wide range of species, including those that are difficult to transform. Because of these benefits, TILLING is used not only on agriculturally important crops such as maize, rice, wheat, barley, soybean, and tomato, but also on animals such as *Drosophila* and zebrafish [4,5,6,7,8,9,10]. In TILLING, chemical mutagenesis induces single nucleotide polymorphisms (SNPs) to generate new alleles. SNPs are identified within the target gene by screening the mutant population, often using mismatch-specific endonucleases. Because TILLING screening can be performed easily, rapidly, and inexpensively in the laboratory, anyone can start with the preparation of a mutant population.

We have tried to establish the barley TILLING system using ‘Mannenboshi’, which has high yield and good quality grains but is susceptible to fungal pathogens, e.g., powdery mildew pathogen *Blumeria graminis* f. sp. *hordei* (*Bgh*) and FHB pathogen *Fusarium graminearum*. This cultivar does not have resistance genes such as *Mla* and is susceptible to most races of *Bgh*. In barley and wheat, there are recessive genes that contribute to disease resistance, and they have become targets for mutagenesis through genome editing. For example, recessive mutations in the *Mlo* gene confer broad-spectrum resistance to *Bgh* [11]. The *mlo* mutation has already been utilized in actual breeding and is valued as a sustainable genetic resource in combination with race-specific resistance genes. In addition to *mlo*, a recessive mutation contributing to FHB resistance is beginning to be found in wheat [12], and these so-called ‘susceptible’ genes are expected to be a powerful tool for resistance breeding because there is no need to introduce foreign genes through transformation. Thus, not only the use of gain-of-function mutations for breeding, but also loss-of-function mutations will contribute as genetic tools to support basic research for genome editing in wheat.

In this study, to establish a barley TILLING system, we prepared a mutant population of 8043 lines. Taking advantage of the fact that the waxy phenotype is derived from a recessive mutation in the *Waxy* gene and is easy to find and non-lethal, we evaluated the availability of the mutant population through a simple and rapid screening of waxy mutants. Sequencing of the *Waxy* gene region in the isolated mutants revealed that the SNPs were novel alleles. Finally, we were able to detect the mutation with the mismatch endonuclease CELI, demonstrating that we can rapidly isolate mutants at such a frequency from our TILLING system.

## 2. Results

### 2.1. Screening for Waxy Mutants from the TILLING Population to Evaluate the Efficiency of Mutant Isolation

**Figure 1:**
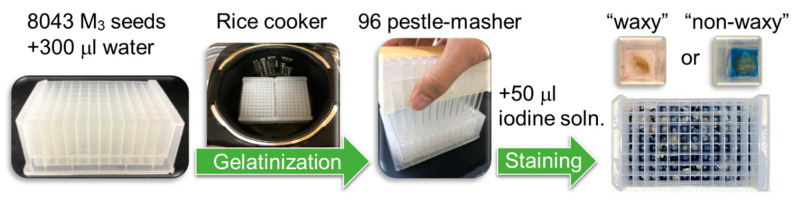
High-throughput screening of waxy mutants from TILLING (Targeting Induced Local Lesions IN Genomes) population. One grain of individual M_3_ seed is placed in each well of 96 deep-well plates and 300 mL of water is added. Seeds are gelatinized with a commercial rice cooker and immediately mashed with a bench-made 96 pestle-masher. After cooled to room temperature, 50 μL of iodine solution is added. The seeds of waxy mutants turn reddish-purple.

After three years of preparation, we obtained 8043 M_3_ seeds of the six-row hull-less barley cultivar ‘Mannenboshi’. To confirm the availability of the TILLING lines, we tested the frequency of mutant occurrence using easily visualized phenotypes as a marker. Although chlorosis is often used as a visible marker, it was not possible to accurately count them because many of them died soon after the first leaf developed due to various environmental stresses in the field, making it impossible to determine whether they were chlorotic or not. Therefore, we focused on the waxy phenotype as a stable phenotype. The mutant seeds exhibiting low amylose content were screened using the iodine-starch reaction. When bound to iodine, amylose molecules turn blue and amylopectin molecules turn reddish-purple. In unpolished seeds, however, the seed coat interfered with iodine staining. Since amylose is easily eluted by gelatinizing the starch grains, mashing the seeds after boiling facilitates the staining of amylose. Therefore, the M_3_ seeds in the 96 deep-well plates were boiled using a commercial rice cooker, mashed with a bench-made 96 pestle-masher, and stained with iodine solution (Figure 1). As a result, four mutants showing reddish-purple were isolated (EUM387, EUM1185, EUM5466, EUM5676).

### 2.2. Sequence Analysis of the Waxy Gene in the Isolated Mutants

**Figure 2:**
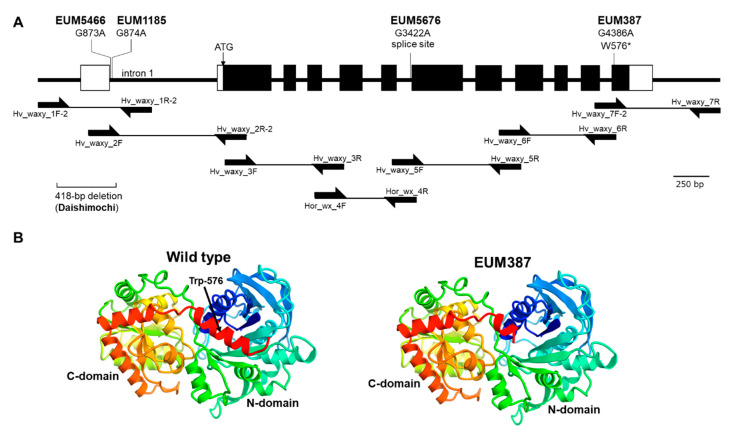
Positions of the identified single nucleotide polymorphisms (SNPs) in the *Waxy* gene. (**A**) The white and black boxes represent the UTRs and the coding regions, respectively. The nucleotide numbers refer to Domon et al., (2002). The primers and the sequenced PCR amplicons are shown as the arrows and the lines. The positions of SNPs identified from the isolated mutants are shown. EUM5466 and EUM1185 have base substitutions of G873A and G874A in the intron 1, respectively. EUM5676 has a base substitution of G3422A at the 3′ splice site of the intron 7. EUM387 has a base substitution of G4386A, which generates a stop codon (*). ‘Daishimochi’ used as a positive control for screening lacks 5′-UTR due to a deletion of 418 bp. (**B**) The barley GBSSI was modeled by Swissmodel using the catalytic domain of rice GBSSIa as a template (PDB ID 3VUF). The C-terminal helix containing Trp-576, which is in close proximity to the N-domain comprising the active center, is lost in EUM387.

Granule-bound starch synthase (GBSS1) is the main enzyme involved in amylose synthesis, and the loss of the function results in a decrease in amylose content in barley [13]. GBSS1 is encoded by the *Waxy* gene. The coding region of the *Waxy* gene was amplified from genomic DNA extracted from the wild type and each mutant. Due to the lack of the genomic information on ‘Mannenboshi’, primer sets were selected and made with reference to Asare et al. [13] and Domon et al. [14] (Table 1), and seven fragments covering the coding region were amplified under PCR conditions that yielded a single band and sequenced (Figure 2A). The sequence of the 5′ upstream noncoding (position 1–451) and the 3′ downstream noncoding (position 4653–5190) regions were not obtained, although the coding region was 99.976% identical to that of the Shikoku Hadaka No.84 (accession AB088761) [14]. The waxy mutant EUM387 had a base substitution of a G to A in the exon 12 (position 4386), which converting Trp-576 of the wild-type gene into a stop codon. EUM5676 had a base substitution of a G to A at the 3′ splice site of the intron 7 (position 3422). EUM5466 and EUM1185 had base substitutions of G to A in the intron 1 (position 873 and 874), respectively.

### 2.3. Detection of a Mutation in the Waxy Gene by Cleavage of Heteroduplex DNA Using the Purified Recombinant CELI Endonuclease

**Figure 3:**
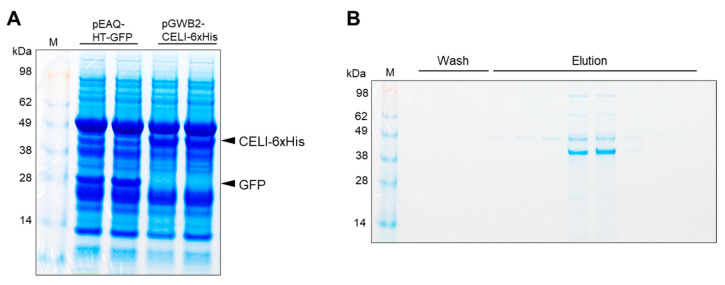
Expression of recombinant CELI protein by an agrobacterium-based transient expression in *Nicotiana benthamiana*. (**A**) SDS-PAGE analysis of the recombinant CELI protein extracted from the leaves after 5 d of agroinfiltration. (**B**) The affinity purification of CELI-6xHis protein using a nickel column.

**Figure 4:**
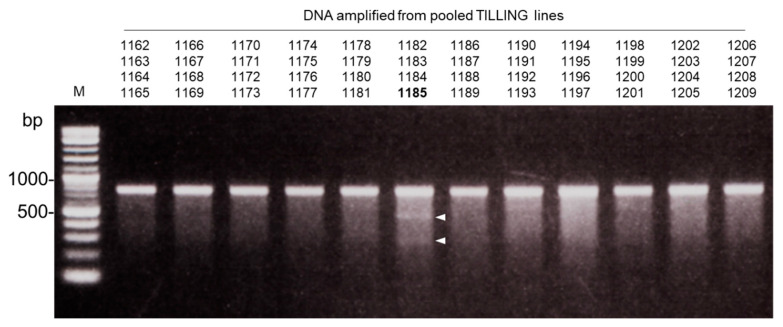
Detection of a mutation in the *Waxy* gene by cleavage of heteroduplex DNA using the purified recombinant CELI endonuclease. The *Waxy* gene fragments amplified from mixed genomic DNA of four individual TILLING lines around EUM1185 were treated with the recombinant CELI protein. Arrowheads indicate two cleaved bands derived from EUM1185.

In general, when detecting mutations in the TILLING system, heteroduplex DNA is cleaved using CELI endonuclease extracted directly from celery plants (*Apium graveolens*) [4]. However, it is necessary to prepare several kilograms of celery plants because the extraction efficiency is very low, and it is also difficult to adjust it as an enzyme solution with consistent activity at all times [15]. In addition, active recombinant CELI protein cannot be expressed in prokaryotes [16]. Therefore, recombinant proteins were synthesized by an agrobacterium-based transient expression method in *Nicotiana benthamiana*. SDS-PAGE analysis of the proteins extracted from the leaves after 5 d of agroinfiltration showed a band of about 43 kDa band of CELI-6xHis protein at a strong level (Figure 3A). In contrast, about 28 kDa band of GFP protein was found in proteins extracted from leaves agroinfiltrated with pEAQ-HT-GFP [17], but not about 43 kDa, suggesting that it was possible to express the recombinant CELI-6xHis protein in *N. benthamiana*. Since the 6xHis tag was added to the C-terminus of CELI, the affinity purification was performed using a nickel column. About 43 kDa CELI protein was eluted, although there was still a band that appeared to be RuBisco (Figure 3B). Two fractions containing the recombinant CELI protein were used to cleave the heteroduplex DNA. To check whether the waxy mutation in EUM1185 could be detected, the *Waxy* gene fragments amplified from mixed genomic DNA of four individual TILLING lines were purified and treated with the recombinant CELI protein. Two cleaved bands appeared only in the lane containing DNA from EUM1185 (Figure 4). The results suggest that our method can detect mutations and that the TILLING population developed in this study will contribute as a fundamental material for barley reverse genetics studies.

## 3. Discussion

The TILLING system is usually developed using M_2_ or M_3_ generation populations. When trying to develop on a large-scale screening system using practical crops rather than model plants that can be grown in the laboratory, it is necessary to carefully consider whether or not we have been able to mutate them to achieve the expected frequency of mutation. In the case of cereal crops such as barley, the approximate frequency of mutations can be estimated by examining the waxy phenotype of the seeds before starting screening. Therefore, in this study, we have devised a high-throughput method of staining starch with iodine solution. The waxy mutants were found very easily and quickly by simply mashing the boiled seeds with a bench-made 96 pestle masher. Even if 8000 individual seeds have to be analyzed, the screening process can be completed in 10 days because 8 plates can be done in a day. Besides, the method saves seeds of mutant populations because only one seed is needed to determine whether it shows the waxy phenotype. One thing to keep in mind is that the method only selects homozygous mutants because the *Waxy* gene is a single recessive gene. Therefore, since the TILLING system can also detect heterozygous mutants, it would be necessary to estimate that there are more mutations in the population.

Several waxy cultivars have been characterized, mostly due to deletions or SNPs in the region of *Waxy* gene [13,14,18]. In this study, sequence analysis of the gene of the isolated mutants revealed four novel alleles carrying SNPs. One of them, EUM387, had a mutation in the coding region, with Trp-576 converted to a stop codon. This suggests that the GBSSI is translated with a truncated C-terminal region consisting of 33 amino acids at the end. This region is different from the catalytic site (residues 81–567) [19]. However, according to the rice GBSSI protein structure reported by Momma and Fujimoto [20], the helix of the C-terminal region (residues 569–585) may form a catalytic center together with the N-terminal domain (residues 83–355) and the C-terminal domain (residues 356–568). Trp-576 is located in the C-terminal helix of barley GBSSI (residues 568-584; SWKGPAKNWEDVLLELG), and the mutation to the stop codon may cause a deletion on the C-terminal half of the helix, causing a decrease in the activity. The structure modeled using the catalytic domain of rice GBSSIa as a template (PDB ID 3VUF) shows that the C-terminal helix containing Trp-567 is in close proximity to the domain comprising the active center, strongly suggesting that the C-terminal truncation by the mutation may affect the activity (Figure 2B). In EUM5676, a base substitution of a G to A occurred at the splice site just before the exon 8. The base substitution creates a frameshift by forming a new splice site along with the next G, resulting in a stop codon after 18 bp. This prevents the formation of an active center because almost only the N-terminal domain is translated. Alternatively, nonsense mediated mRNA decay may prevent the synthesis of abnormal proteins in advance [21,22]. On the other hand, the mutations in EUM5466 and EUM1185 are located at the 5′ side of the intron 1 within the 5′-UTR, and thus seem not to be associated with nonsense and frameshift mutations, as well as missense mutations. Interestingly, in the parental line of the waxy cultivar ‘Daishimochi’, ‘Mochimugi D’, there is a 418 bp deletion in the 5′-UTR (accession AB087716) [14], and these bases are located in the deleted region. It will be necessary to determine whether an intron in the noncoding region affects the accumulation of mRNA or protein.

Although the mutation density cannot be easily compared between populations, as it varies with the type, the concentration, and the treatment time of mutagens, it was estimated in the TILLING populations of several barley cultivars (about one mutation per 1000 kb in ‘Optic’ [6] and about one per 374 kb in ‘Morex’ [23]). In the ‘Mannenboshi’ TILLING population, 4 mutations were identified by screening 8043 lines. Since the sequenced region length of *Waxy* gene was 4200 bp, the mutation density is tentatively estimated as 4 mutations/(4200 bp × 8043 lines), i.e., one mutation per 8445 kb. However, in fact, 4 mutations were found in the *Waxy* gene as homozygous recessive mutations by forward genetics, and a TILLING screening would probably detect more silent mutations and mutations in the non-coding region that do not contribute to the phenotype, as well as heterozygous mutations. In the report by Talamè et al. [23], the estimated mutation density was about one mutation per 548 kb when only missense mutations were calculated. In addition, 15 missense mutations were found in four genes, which means that about 4 missense alleles can be found per gene. Presumably, our TILLING population has a similar mutation density as that of Talame et al. Recently, a TILLING population of ‘Golden Promise’ was reported [24]. Because of the very high mutation density (one mutation per 154 kb) due to repeated mutagen treatments, it is possible that mutations in the gene of interest as well as in other genes could affect the phenotype, but if a line with mutations in the *Mla8* gene can be obtained, it would be a very powerful tool for analyzing defense responses to *Bgh*, as transformation is possible in ‘Golden Promise’.

A rapid and low-cost method is required to screen from a large scale mutant population of about 8000 lines. CELI is widely used as an effective and cost-effective enzyme for detecting mismatch sites. However, as pointed out by Yao et al. [25], it has to be extracted from a large amount of celery and the extraction efficiency is very low. Therefore, we tested whether heteroduplex DNA could be cleaved by CELI which were expressed in *N. benthamiana* and affinity-purified. The results showed that even a simple purification, in which RuBisco was still present, was active enough. Even two leaf discs (5 mm in diameter) showed sufficient accumulation at the SDS-PAGE level, indicating that the enzyme could be prepared efficiently at low cost. After the affinity purification from 1 g leaf sample, the CELI solution for more than 2000 reactions was obtained. Recently, Yao et al. were also able to express CELI protein in *N. benthamiana*, but they were only able to detect it at the Western blotting level [25]. They may be able to express a large amount of CELI protein if they use the *Agrobacterium tumefaciens* strain enabling high expression (C58 strain) as we did. However, even with our CELI production method, it would be better to purify more by gel filtration and develop a buffer with stable activity. Since there were reports of better cleavage efficiency when Mg^2+^ was added to the reaction buffer than Ca^2+^ or Mn^2+^ [25] and that Ca^2+^ and Sr^2+^ were more effective in CELII, another endonuclease [26]. However, since there was not much difference in our system, we decided to use Mg^2+^. In addition, although it was a preliminary experiment, the purified heteroduplex DNA fragments could be cleaved cleanly, but the heteroduplex DNA fragments in the PCR reaction solution were difficult to be cleaved by direct CELI treatment. In fact, we realize that there is a need to choose a DNA polymerase and a reaction buffer, and Takara Ex taq was the best of all the ones we tested. Needless to say, the selection of primer sets to amplify the single band is also necessary. Furthermore, it is important to keep in mind that mixing more than six genomic DNA may reduce the ratio of mutant fragments and thus make them harder to detect. Taken together, we may have to start using the next generation sequencing technologies that can be performed in a single laboratory at a reasonable cost, such as Oxford Nanopore Technologies’ MinION, to analyze amplicons in a high throughput manner.

Genomic DNA has now been extracted from most of the 8043 mutants, and TILLING screening will soon begin in our laboratory to reverse-genetically isolate mutants involved in the plant immunity. Because of the current abundance of useful information and technologies, even a small research group such as ours can now prepare mutant populations of not only model plants but also crops, isolate more and more mutants, and utilize them as genetic tools for basic research. In the near future, the technology of genome editing will be increasingly advanced. The development of wheat and other major crops directly related to human life will be accelerated by the technology. To support this, we strongly believe that it is important to develop barley, which is genetically most closely related to wheat, as a new model plant such as *Arabidopsis* and rice.

## 4. Materials and Methods

### 4.1. Plant Materials and Mutagenesis

The six-row hull-less barley cultivar ‘Mannenboshi’ seeds were treated with 0.5 mM sodium azide in potassium phosphate buffer (pH3.0) for 2 h to make M_1_ seeds. After the treatment, the seeds were washed five times with deionized water. About 20,000 M_1_ seeds were sown in December 2016. M_2_ seeds were obtained from each of the 11,388 surviving fertile plants and were sown individually in November 2017. M_3_ seeds were obtained from 8043 surviving fertile plants, numbered individually and stored at 15 °C as a mutant population. A waxy cultivar ‘Daishimochi’ was used as a positive control for iodine staining screening.

### 4.2. Iodine Staining 

To visualize amylose content in a high-throughput manner, one grain of individual seed was stained with 1 mM iodine solution (0.25 g iodine, 1.2 g potassium iodide, 1 liter water) in 96 deep-well plates (2 mL/well). Because it was difficult to determine if unpolished seeds were stained or not with iodine solution, seeds were gelatinized with a commercial rice cooker and mashed with a bench-made 96 pestle-masher (Figure 1). Square acrylic sticks were inserted into each well of the same deep-well plate and immobilized with glue to make the 96 pestle-masher. After boiling in the well containing 300 µL of water, the seeds were immediately mashed, cooled to room temperature, and stained with 50 µL of iodine solution.

### 4.3. Genomic DNA Extraction

Genomic DNA was extracted from 7-day-old barley seedlings grown in vermiculite supplemented with 300-fold diluted HYPONeX (N:P:K = 6:10:5, Hyponex Japan, Osaka, Japan) in a growth chamber (NK system LH-200-RD, Nippon Medical & Chemical Instruments Co., Ltd., Osaka, Japan) at 20 °C under the continuous light condition as described in Wahara et al. [27]. Approximately 2 cm^2^ leaf segments were sampled in a 2 mL screw-cap tube containing two zirconia beads and frozen in liquid nitrogen. The tubes were placed in an aluminum block cooled with liquid nitrogen, set in the Shake Master Neo (BMS-M10N21, Biomedical Science Co., Ltd., Tokyo, Japan) and powdered for 60 s at 1500 rpm. Then, 600 µL of the DNA extraction buffer (1% (*w*/*v*) N-lauroyl sarcosyl sodium salt, 100 mM Tris, 100 mM NaCl, 0.32% (*w*/*v*) Na_2_EDTA, and 2% (*w*/*v*) PVP) and 6 µL of 10 mg/mL RNase A were added and vortexed vigorously. The extracts were incubated for 1 h at 37 °C with vortexing every 20 min. Equal amounts of PCI (phenol: chloroform: isoamyl alcohol = 25:24:1) were added and mixed for 15 min using a rotator (RT-50, TAITEC Co., Ltd., Koshigaya, Japan). The samples were then centrifuged at 14,800 rpm for 10 min at room temperature. The supernatant was transferred to a new 1.5 mL tube and equal amounts of CI (chloroform: isoamyl alcohol = 24:1) were added and mixed by inversion for 10 min. After centrifugation at 14,800 rpm for 10 min at room temperature, the supernatants were mixed with 3 M sodium acetate (pH4.8) and 2-propanol in a new 1.5 mL tube. After precipitation with centrifugation, the pellets were washed with 70% (*v*/*v*) ethanol and dried in a vacuum. Then, extracted genomic DNA was dissolved in Tris-EDTA buffer and stored at −30 °C.

### 4.4. Amplification and Sequencing of the Waxy Gene Fragments

Seven fragments of the *Waxy* gene were amplified from the extracted genomic DNA using the primer sets (Table 1). The PCR was performed in a 20 µL reaction mixture containing ~100 ng genomic DNA, 0.1 µM of each primer, 200 µM dNTP, 1.5 mM MgSO_4_, 1× PCR buffer, and 1 unit of KOD -Plus- neo polymerase (TOYOBO Co., Ltd., Osaka, Japan). PCR with primer sets (Hv_waxy_5F/Hv_waxy_5R and Hv_waxy_6F/Hv_waxy_6R) was performed in a three-step cycle (pre-incubation at 98 °C for 2 min, followed by 35 cycles of 98 °C for 30 s, 55 °C for 30 s, 72 °C for 1 min, and a final extension at 72 °C for 1 min. Step-down thermal cycling consisted of a pre-incubation at 94 °C for 2 min, 5 cycles of 98 °C for 10 s and 74 °C for 1 min, 5 cycles of 98 °C for 10 s and 72 °C for 1 min, 5 cycles of 98 °C for 10 s and 70 °C for 1min, 20 cycles of 98 °C for 10 s and 68 °C for 1 min, and a final extension at 68 °C for 7 min was performed using primer sets (Hv_waxy_1F-2/Hv_waxy_1R-2, Hv_waxy_2F/Hv_waxy_2R-2, Hv_waxy_3F/Hv_waxy_3R, Hor_wx_4F/Hor_wx_4R and Hv_waxy_7F-2/Hv_waxy_7R). After electrophoresis on an agarose gel, the bands of each gene fragment were cut out, and the gene fragments were extracted using a gel extraction kit (FastGene Gel/PCR Extraction Kit, Nippon Genetics Co., Ltd., Tokyo, Japan). Sanger sequence was performed using the primers (Table 1).

### 4.5. Cleavage of Heteroduplex DNA Using a Recombinant CELI Endonuclease

Endonuclease CELI was used to detect mutations [15]. First, the construct was generated for expression in *N. benthamiana* leaves. The fragment of CELI gene was amplified with a primer set (infu_CELI; GCCGCCCCCTTCACCATGACGCGATTATATTCTGTGTTCTTTCT/CEL-6xHis_infu; GGCGCGCCCACCCTTTCAGTGGTGGTGGTGGTGGTGTTCTTCTGCCAAA) using artificially synthesized DNA as a template. After purification from the agarose gel, the fragments were introduced into the linearized pENTR vector (Thermo Fisher Scientific Inc., Waltham, MA, USA) using the In-Fusion HD Cloning Kit (Takara Bio Inc., Suozu, Shiga Prefecture, Japan). The resulting pENTR-CELI-6xHis and pGWB2 vector [28] were LR-reacted to generate pGWB2-CELI-6xHis. After preparation of the plasmid from *E. coli* (strain DH5α), *A. tumefaciens* (strain C58) was transformed with it. To express the CELI protein, the leaves of *N. benthamiana* were inoculated with the mixtures of agrobacteria harboring pGWB2-CELI-6xHis and the P19 silencing suppressor as described previously [29]. The inoculated leaves were sampled 5 d after agroinfiltration. To check the expression, two leaf discs (5 mm in diameter) were powdered using a bead shocker (ShakeMaster NEO, BMS-M10N21, Bio Medical Science, Tokyo, Japan) and heated at 95 °C for 5 min after 2× Laemmli sample buffer was added. After centrifugation, the supernatant was separated by SDS-PAGE. For purification, about 1 g leaves were ground with a mortar and pestle in liquid nitrogen. The extraction buffer (25 mM Tris-HCl pH7.0, 50 mM NaCl, 2 mM DTT) was added and the supernatants were collected after centrifugation. The extracts were filtrated with a 0.45 µm PES filter and purified through the nickel-charged Sepharose column (HisTrap HP, GE Healthcare, Uppsala, Sweden). Defining the CELI activity required to cleave heteroduplex DNA fragments (about 10 ng) as one unit, more than 2000 units were obtained. The CELI solution was adjusted to 1 unit/µL. The heteroduplexes were prepared with the amplified DNA fragments (a primer set; Hv_waxy_1F-2 and Hv_waxy_1R-2) at 99 °C for 10 min (denature) and 70 °C for 20 s (annealing). The mixtures of the heteroduplex DNA and the recombinant CELI protein were incubated in the reaction buffer (1 mM HEPES pH7.5, 5 mM KCl, 1 mM MgSO_4_, 0.0002% Triton X-100, 200 ng/mL BSA) at 45 °C for 30 min. The cleavage was confirmed by ethidium bromide-stained agarose gel electrophoresis.

## Figures and Tables

**Table 1:** Primers used for the *Waxy* gene amplification.

Primer	Sequence (5′→3′)	Direction
Hv_waxy_1F-2	CTTCATCTTCCTCCTGTCCTGTGTG	Forward
Hv_waxy_1R-2	AGCCGTACGTTGGATCTGTTCCTGAAA	Reverse
Hv_waxy_2F	ACACACTACAACCTCTGCCACT	Forward
Hv_waxy_2R-2	TTGCTCCGACAGTCCTCATACC	Reverse
Hv_waxy_3F	TCTGGCCACGTCCCAGCT	Forward
Hv_waxy_3R	TCTTCTCCTTGGTCTTGCCCC	Reverse
Hor_wx_4F	TACAAGCGCGGAGTGGAC	Forward
Hor_wx_4R	ACGAGATGTTGTGGATGCAG	Reverse
Hv_waxy_5F	AGTCCAATGGCATCTACAGG	Forward
Hv_waxy_5R	CTCTTGAGCAGCTTCTCAAAC	Reverse
Hv_waxy_6F	GACGTCCAGATCATTCTCCTT	Forward
Hv_waxy_6R	ACGTCCTCCCAGTTCTTGGCA	Reverse
Hv_waxy_7F-2	GGTCAAGAACTGCATGATCCAGGAT	Forward
Hv_waxy_7R	TGTTGCATCGATCTTGGCG	Reverse

## References

[1] Hisano H., Sato K. (2016). Genomic regions responsible for amenability to Agrobacterium-mediated transformation in barley. Sci. Rep..

[2] McCallum C.M., Comai L., Greene E.A., Henikoff S. (2000). Targeted screening for induced mutations. Nat. Biotechnol..

[3] Till B.J., Cooper J., Tai T.H., Colowit P., Greene E.A., Henikoff S., Comai L. (2007). Discovery of chemically induced mutations in rice by TILLING. BMC Plant Biol..

[4] Till B.J., Reynolds S.H., Weil C., Springer N., Burtner C., Young K., Bowers E., Codomo C.A., Enns L.C., Odden A.R. (2004). Discovery of induced point mutations in maize genes by TILLING. BMC Plant Biol..

[5] Uauy C., Paraiso F., Colasuonno P., Tran R.K., Tsai H., Berardi S., Comai L., Dubcovsky J. (2009). A modified TILLING approach to detect induced mutations in tetraploid and hexaploid wheat. BMC Plant Biol..

[6] Caldwell D.G., McCallum N., Shaw P., Muehlbauer G.J., Marshall D.F., Waugh R. (2004). A structured mutant population for forward and reverse genetics in barley (*Hordeum vulgare* L.). Plant J..

[7] Cooper J.L., Till B.J., Laport R.G., Darlow M.C., Kleffner J.M., Jamai A., El-Mellouki T., Liu S., Ritchie R., Nielsen N. (2008). TILLING to detect induced mutations in soybean. BMC Plant Biol..

[8] Minoia S., Petrozza A., D’Onofrio O., Prion F., Mosca G., Sozio G., Cellini F., Bendahmane A., Carriero F. (2010). A new mutant genetic resource for tomato crop improvement by TILLING technology. BMC Res. Notes..

[9] Winkler S., Schwabedissen A., Backasch D., Bökel C., Seidel C., Bönisch S., Fürthauer M., Kuhrs A., Cobreros L., Brand M. (2005). Target-selected mutant screen by TILLING in *Drosophila*. Genome Res..

[10] Wienholds E., van Eeden F., Kosters M., Mudde J., Plasterk R.H., Cuppen E. (2003). Efficient target-selected mutagenesis in zebrafish. Genome Res..

[11] Büschges R., Hollricher K., Panstruga R., Simons G., Wolter M., Frijters A., van Daelen R., van der Lee T., Diergaarde P., Groenendijk J. (1997). The barley *Mlo* gene: A novel control element of plant pathogen resistance. Cell.

[12] Su Z., Bernardo A., Tian B., Chen H., Wang S., Ma H., Cai S., Liu D., Zhang D., Li T. (2019). A deletion mutation in *TaHRC* confers *Fhb1* resistance to Fusarium head blight in wheat. Nat. Genet..

[13] Asare E.K., Baga M., Rossnagel B.G., Chibbar R.N. (2012). Polymorphism in the barley granule bound starch synthase 1 (*Gbss1*) gene associated with grain starch variant amylose concentration. J. Agri. Food Chem..

[14] Domon E., Saito A., Takeda K. (2002). Comparison of the waxy locus sequence from a non-waxy strain and two waxy mutants of spontaneous and artificial origins in barley. Genes Genet. Syst..

[15] Yang B., Wen X., Kodali N.S., Oleykowski C.A., Miller C.G., Kulinski J., Besack D., Yeung J.A., Kowalski D., Yeung A.T. (2000). Purification, cloning, and characterization of the CEL I nuclease. Biochem..

[16] Pimkin M., Caretti E., Canutescu A., Yeung J.B., Cohn H., Chen Y., Oleykowski C., Bellacosa A., Yeung A.T. (2007). Recombinant nucleases CEL I from celery and SP I from spinach for mutation detection. BMC Biotechnol..

[17] Sainsbury S., Thuenemann E.C., Lomonossoff G.P. (2009). pEAQ: Versatile expression vectors for easy and quick transient expression of heterologous proteins in plants. Plant Biotechnol. J..

[18] Patron N.J., Smith A.M., Fahy B.F., Hylton C.M., Naldrett M.J., Rossnagel B.G., Denyer K. (2002). The altered pattern of amylose accumulation in the endosperm of low-amylose barley cultivars is attributable to a single mutant allele of granule-bound starch synthase I with a deletion in the 5’-non-coding region. Plant Physiol..

[19] Hebelstrup K.H., Nielsen M.M., Carciofi M., Andrzejczak O., Shaik S.S., Blennow A., Palcic M.M. (2017). *Waxy* and *non-waxy* barley cultivars exhibit differences in the targeting and catalytic activity of GBSS1a. J. Exp. Bot..

[20] Momma M., Fujimoto Z. (2012). Interdomain disulfide bridge in the rice granule bound starch synthase I catalytic domain as elucidated by X-ray structure analysis. Biosci. Biotechnol. Biochem..

[21] Isshiki M., Yamamoto Y., Sato H., Shimamoto K. (2001). Nonsense-mediated decay of mutant waxy mRNA in rice. Plant Physiol..

[22] Chang Y.F., Imam J.S., Wilkinson M.F. (2007). The nonsense-mediated decay RNA surveillance pathway. Ann. Rev. Biochem..

[23] Talamè V., Bovina R., Sanguineti M.C., Tuberosa R., Lundqvist U., Salvi S. (2008). TILLMore, a resource for the discovery of chemically induced mutants in barley. Plant Biotechnol. J..

[24] Schreiber M., Barakate A., Uzrek N., Macaulay M., Sourdille A., Morris J., Hedley P.E., Ramsay L., Waugh R. (2019). A highly mutagenised barley (*cv*. Golden Promise) TILLING population coupled with strategies for screening-by-sequencing. Plant Methods.

[25] Yao X.F., Wu S., Guo L., Liu C.M. (2020). Efficient CELI endonuclease production in *Nicotiana benthamiana* through transient expression and applications in detections of mutation and gene editing events. Plant Sci..

[26] Mon H., Lee J., Fukushima M., Nagata Y., Fujii M., Xu J., Nishi O., Iiyama K., Kusakabe T. (2013). Production and characterization of celery mismatch endonuclease CEL II using baculovirus/silkworm expression system. Appl. Microbiol. Biotechnol..

[27] Wahara M., Inoue C., Kohguchi T., Sugai K., Kobayashi K., Nishiguchi M., Yamaoka N., Yaeno T. (2017). Improved method for in situ biolistic transformation to analyze barley-powdery mildew interactions. J. Gen. Plant Pathol..

[28] Nakagawa T., Kurose T., Hino T., Tanaka K., Kawamukai M., Niwa Y., Toyooka K., Matsuoka K., Jinbo T., Kimura T. (2007). Development of series of gateway binary vectors, pGWBs, for realizing efficient construction of fusion genes for plant transformation. J. Biosci. Bioeng..

[29] Yaeno T., Li H., Chaparro-Garcia A., Schornack S., Koshiba S., Watanabe S., Kigawa T., Kamoun S., Shirasu K. (2011). Phosphatidylinositol monophosphate-binding interface in the oomycete RXLR effector AVR3a is required for its stability in host cells to modulate plant immunity. Proc. Natl. Acad. Sci. USA.

